# Comparison of Neutron Detection Performance of Four Thin-Film Semiconductor Neutron Detectors Based on Geant4

**DOI:** 10.3390/s21237930

**Published:** 2021-11-27

**Authors:** Zhongming Zhang, Michael D. Aspinall

**Affiliations:** Engineering Department, Lancaster University, Lancaster LA1 4YW, UK; m.d.aspinall@lancaster.ac.uk

**Keywords:** interaction of radiation with matter, neutron detection, semiconductor, charge collection efficiency, radiation-hard detectors, Geant4, Monte Carlo simulation

## Abstract

Third-generation semiconductor materials have a wide band gap, high thermal conductivity, high chemical stability and strong radiation resistance. These materials have broad application prospects in optoelectronics, high-temperature and high-power equipment and radiation detectors. In this work, thin-film solid state neutron detectors made of four third-generation semiconductor materials are studied. Geant4 10.7 was used to analyze and optimize detectors. The optimal thicknesses required to achieve the highest detection efficiency for the four materials are studied. The optimized materials include diamond, silicon carbide (SiC), gallium oxide (Ga${}_{2}$O${}_{3}$) and gallium nitride (GaN), and the converter layer materials are boron carbide (B${}_{4}$C) and lithium fluoride (LiF) with a natural enrichment of boron and lithium. With optimal thickness, the primary knock-on atom (PKA) energy spectrum and displacements per atom (DPA) are studied to provide an indication of the radiation hardness of the four materials. The gamma rejection capabilities and electron collection efficiency (ECE) of these materials have also been studied. This work will contribute to manufacturing radiation-resistant, high-temperature-resistant and fast response neutron detectors. It will facilitate reactor monitoring, high-energy physics experiments and nuclear fusion research.

## 1. Introduction

Gas detectors, scintillator detectors and semiconductor detectors are the most common detectors for neutron detection. Gas detectors and scintillator detectors have a high neutron detection efficiency and good time response [1,2]. However, they are relatively sensitive to gamma rays, and their sensitive volumes are more significant than semiconductor detectors. Although semiconductor neutron detectors have a relatively low detection efficiency, their small size, fast response and insensitivity to gamma rays make them a good candidate for neutron detection under extreme environments, especially for wide band gap semiconductors.

In theory, for thin-film semiconductor neutron detectors, the detection efficiency will increase as the thickness of the semiconductor increases. However, the self-absorption effect of the converter layer will make the situation more complicated. Furthermore, according to the Hecht equation, a thicker semiconductor will decrease the charge collection efficiency (CCE). To find the optimal thickness for neutron detection, the diode thicknesses for Si, C, GaAs and CdTe thin-film solid state neutron detectors were optimised [3].

However, the development of nuclear fusion, new generation nuclear fission reactors and outer space nuclear technology is inseparable from neutron detection under extreme environments. The environments that such scenarios impose on neutron detectors are complex and extreme, presenting high-intensity radiation fields, high temperatures and high pressures, as well as being highly corrosive. More advanced neutron detectors that can withstand use in extreme environments are required. Si, GaAs and CdTe do not meet these requirements.

Diamond detectors have a wide band gap (5.5 eV), high melting point (4373 ${}^{\circ }$C at 127 kbar), strong radiation resistance and one hundred hours service life under high neutron flux [4,5]. Radiation resistance studies of chemical vapor deposition (CVD) diamond, which irradiate the material with photons, protons, pions and alpha particles, demonstrate that its signal collection properties do not change with photons up to 10 Mrad, protons up to 5 × 10${}^{13}$ cm${}^{-2}$ and pions up to 8 × 10${}^{13}$ cm${}^{-2}$ [6]. They indicate that diamond detectors could be used in an extreme environment with a practical service life. In 2009, a single-crystal diamond-based thermal neutron beam monitor was applied on the Italian Neutron Experimental Station (INES) beam line at the ISIS spallation neutron source (Great Britain) [7]. It demonstrated the thermal neutron detection monitoring ability of diamond-based detectors. In 2019, the thermal neutron and gamma detection performance of a diamond detector with a LiF converter layer was studied [8], which further verified the thermal neutron detection capability and gamma rejection capability of the detector. A study of the performance of diamond detectors in a high-temperature environment showed that diamond can work properly in a spectrometric mode at temperatures of up to 240 ${}^{\circ }$C with an energy resolution (FWHM) of approximately 3.5% [9]. In addition to a diamond detector with a LiF converter layer, the thermal neutron detection performance of a PIN-type diamond detector using a boron nitride converter layer was reported [10].

In 2020, a review on SiC detector development was presented [11]. In the same year, a study of the thermal neutron irradiation influence on the structural and electrical properties of n-4H-SiC and n-Al/n-4H-SiC Schottky contacts was reported [12]. This study indicated that neutron irradiation would affect the electrical properties of the 4H-SiC detector, especially for n-Al/n-4H-SiC Schottky contacts. Both Schottky and ohmic contacts are used to improve the performance of a novel SiC-based strip sensor. Alpha particles and tritons can be identified clearly from the spectrum obtained by the detector [13]. SiC can be used as near core neutron flux detectors given its strong radiation resistance capability [14]. Furthermore, a radiation resistance capability comparison between Si and 4H-SiC was conducted [15]. It demonstrated that, due to the lower thickness and high doping level, 4H-SiC is a more reliable device than Si in a radiation environment.

Ga${}_{2}$O${}_{3}$ (β-Ga${}_{2}$O${}_{3}$) is an ultra-wide band gap semiconductor material with a band gap of approximately 4.8 eV, a theoretical breakdown field strength of 8 MV/cm and an electron mobility of 300 cm${}^{2}$/V·s. The Baliga’s figure-of-merit of β-Ga${}_{2}$O${}_{3}$ is 4 times that of GaN, 10 times that of SiC and 3444 times that of Si. In addition, a large-size β-Ga${}_{2}$O${}_{3}$ with low defect density (10${}^{3}$ cm${}^{-2}$ to 10${}^{4}$ cm${}^{-2}$) can be obtained by growing on a sapphire substrate. This makes the cost of β-Ga${}_{2}$O${}_{3}$ devices lower than GaN and SiC devices. In 2019, induced defects in β-Ga${}_{2}$O${}_{3}$ Schottky diodes were studied under high energy neutron radiation [16]. The results demonstrated that high-energy neutron irradiation will reduce the number of carriers and primary effects of neutron irradiation on the conduction band energy (E${}_{c}$), increasing the concentration of a state at E${}_{c}$−2.00 eV and introducing a state at E${}_{c}$−1.29 eV. Research on Ga${}_{2}$O${}_{3}$ deep-level defects presented the relationship between neutron irradiation and defect concentrations and the anneal temperature influence on the defect distribution [17]. Such work will help to control the electric properties of Ga${}_{2}$O${}_{3}$.

GaN is a third-generation semiconductor material with the biggest band gap of all commercial semiconductors [4]. GaN can be grown on a variety of substrates: AlN [18], Si [19], sapphire [20] and SiC [21]. In 2014, a BGaN detector with a good α particle sensitivity and low gamma sensitivity was demonstrated [22], and it showed a good detection performance under high radiation conditions. In 2020, a GaN neutron detector with LiF was fabricated with a neutron detection efficiency of approximately 1.9% under a 0 V bias [23]. There are also studies using GaN for fast neutron detectors [24] and boron neutron capture therapy monitors [25,26]. Besides the layered structure, ion-implanted GaN detectors have also been studied [27]. There is recent research about growing GaN on nitrogen-doped single layer graphene (n-SLG) substrates [28]. These results demonstrated that GaN with high electron contents could be obtained using this method, which will aid the fabrication of superior GaN devices.

This research demonstrated that semiconductor materials, such as diamond, SiC, Ga${}_{2}$O${}_{3}$ and GaN, are emerging as potential neutron detection materials due to their wide band gap, high breakdown voltage, small dark current, fast carrier mobility speed, considerable carrier drift distance and higher thermal conductivity. They are good candidates for neutron detection in extreme environments.

In this research, with a B${}_{4}$C or LiF converter layer, four semiconductor material thicknesses will be optimised in terms of their neutron detection efficiency and gamma resistance capability. Furthermore, radiation hardness and electron collection efficiency will be studied with optimised thicknesses. Research on these materials will help to accelerate the production of new semiconductor neutron detectors and to supply the demands as nuclear technologies evolve.

## 2. Methods

**Figure 1:**
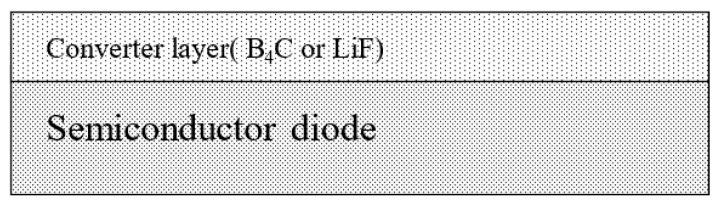
Schematic structure of the simulated detector.

The structure of the detector used during the reported simulations is shown in Figure 1. The active area of the detector is 1 cm${}^{2}$. The detector has two layers: the upper layer is a converter layer composed of B${}_{4}$C or LiF, and the bottom layer is the semiconductor layer. In thermal neutron detection, thermal neutrons will first react with the converter layer to produce charged particles, such as α particles or tritium (T). Charged particles will create electron and hole pairs along its track in the semiconductor. Electrons and holes will drift in the semiconductor with an external electric field, which will induce a current in an external circuit that can be detected. In this study, a dead layer is not added, and the semiconductor material is not ion-implanted, which can be considered as an i-type semiconductor. Therefore, the detection efficiency can be regarded as the intrinsic thermal neutron detection efficiency of the material.

The response of detectors to neutrons is the focus of this research. Geant4 (version 10.7) was used for simulations, along with the FTFP_BERT_HP physics list, which uses the high precision neutron models and cross sections to describe elastic and inelastic scattering, capture and fission for neutrons. The neutron source is placed 1 cm above the geometric center of the detector’s upper surface. The neutron energy for thickness optimization and gamma resistance is 0.025 eV and the neutron energy for radiation hardness research is 1 MeV. The number of neutrons in a single simulation is 5 × 10${}^{7}$ and the number of gammas in a single simulation is 1 × 10${}^{8}$. These quantities were chosen to achieve a small uncertainty whilst maintaining an acceptable simulation runtime. The high-end computing (HEC) cluster at Lancaster University was used for these simulations.

(1) $$N_{\Omega }=N_{Total}\times \frac{\Omega }{4\pi }$$

(2) $$\varepsilon =\frac{Pulses}{N_{\Omega }}$$

The calculation of the absolute detection efficiency of the detector is given by (1) and (2). $N_{\Omega }$ is the number of neutrons or gammas emitted by the source in angle Ω (rad). $N_{Total}$ is the total number of neutrons emitted from the neutron source. ε is the ratio of Pulses recorded by the simulation and the number of source particles in angle Ω ($N_{\Omega }$).

A low-level discriminator (LLD) was introduced to avoid background counting in the actual detection process. Consistent with the literature [29], this study uses an LLD of 300 keV. In addition to this, an LLD of 900 keV was added to observe the influence of different LLDs on the detection efficiency curve.

For different semiconductor neutron detector materials with the same converter layer material and LLD, the optimal thickness of the converter layer should be the same [4]. Therefore, based on prior work, the converter layer materials used during this study were B${}_{4}$C and LiF. The thicknesses of the two converter layers for different LLD simulations are listed in Table 1 [30].

For fixed converter layer thicknesses showed in Table 1, the thickness of the semiconductor layer was optimized by studying the relationship between the semiconductor layer thickness and the detector’s detection efficiency.

The gamma rejection capability of the detector was tested by obtaining the gamma detection efficiency of the detector. This was achieved by replacing the neutron source with a gamma source in the simulations. The gamma detection efficiency of the detector was then calculated using (1) and (2). Through this, the gamma rejection capability of detectors composed of different materials was evaluated.

**Figure 2:**
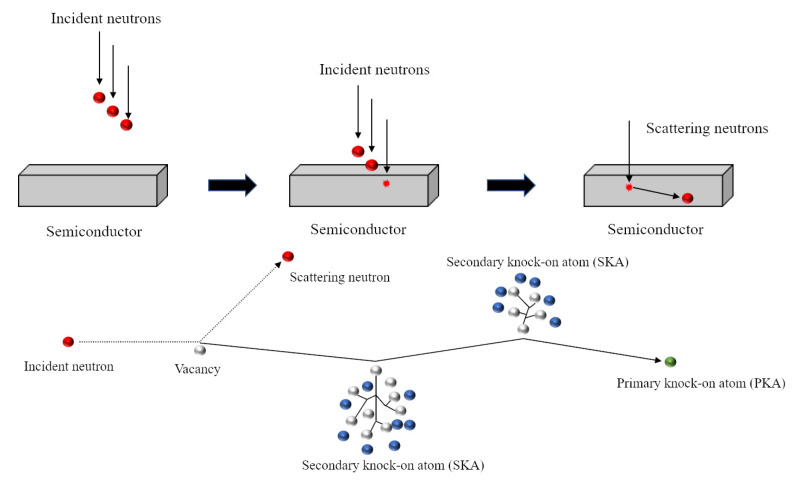
The process of neutron irradiation produces primary knock-on atoms and secondary knock-on atoms in the semiconductor material.

When a neutron collides elastically with an atom within the semiconductor, there is a certain probability that the atom will move and leave the lattice position. An atom that is hit by a neutron and causes it to displace is called a primary knock-on atom (PKA). Since a PKA has part of the kinetic energy of the neutron, it will continue to collide elastically with other atoms. Atoms that collide with PKAs and causes further displacements are called secondary knock-on atoms (SKAs). The process is shown in Figure 2. The accumulation of these displacements can cause material defects. Therefore, calculating the displacements per atom (DPA) can predict the radiation resistance of the material. In this study, we simply simulated the PKA energy spectrum generated by neutron elastic scattering. The thicknesses of semiconductor materials were obtained from thickness optimisation, and semiconductors were irradiated without a converter layer coated. An early model was used to calculate atomic displacements by considering the kinetic energy transfer [31], which is shown in (3).

(3) $$N_{d}\left(T_{d}\right)=\left\{\begin{array}{cc} 0,\;\;\;\;\;\;\;\;\;\;\;\;\;T_{d}\;<\;E_{d} & \\ 1,\;\;\;\;\;\;\;\;\;\;\;\;\;E_{d}\;\leq \;T_{d}\;<\;\frac{2E_{d}}{0.8} & \\ \frac{0.8T_{d}}{2E_{d}},\;\;\;\;\;\frac{2E_{d}}{0.8}\;\leq \;T_{d}\;<\;\infty & \end{array}\right.$$

where $N_{d}$ is the predicted number of atom displacements and $T_{d}$ is the damage energy (eV), which is the kinetic energy transferred to PKA from a neutron in this study. $E_{d}$ is the threshold displacement energy (eV). Table 2 shows the threshold displacement energy for diamond, 4H-SiC, Ga${}_{2}$O${}_{3}$ and GaN.

A 1 MeV neutron flux was used as the particle source in these simulations. In order to perform a qualitative analysis of the radiation hardness of these materials and to simplify the simulations, the threshold displacement energy for GaN was set to 22 eV and the threshold displacement energy for SiC was set to 35 eV.

(4) λ=μ×τ×E

(5) $$\frac{Q}{Q_{0}}=\int_{0}^{x_{d}}\frac{Q_{0}e^{\frac{-x}{\lambda }}}{x_{d}}dx=\frac{\lambda }{x_{d}}\left(1-e^{\frac{-x_{d}}{\lambda }}\right)$$

The charge collection efficiency (CCE) is an important characteristic of semiconductor materials. The single carrier Hecht Equation (5) was used to calculate the electron collection efficiency for four materials at the last part of the research under bias voltage from 5 V to 100 V. In (4), λ is the drift length of the electron, μ is the carrier mobility, which is the electron mobility in this case, τ is the electron lifetime and E is the electric field strength. The value of μ and τ are listed in Table 3. In (5), *Q* is the electron charge collected by the depletion region, $Q_{0}$ is the total electron charge generated by charged particles in the depletion region and $x_{d}$ is the width of the depletion region.

## 3. Results

### 3.1. Thickness Optimisation

**Figure 3:**
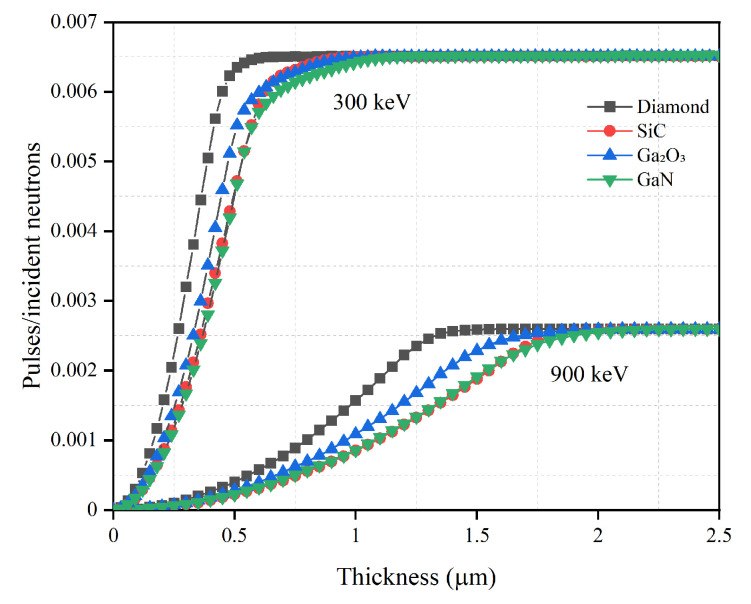
Neutron detection efficiency versus semiconductor material thickness with optimal B${}_{4}$C converter layer thickness for diamond, SiC, Ga${}_{2}$O${}_{3}$ and GaN semiconductor materials. The optimal converter layer thicknesses are 2.6 μm for 300 keV LLD and 1.7 μm for 900 keV LLD, as shown in Table 1.

**Figure 4:**
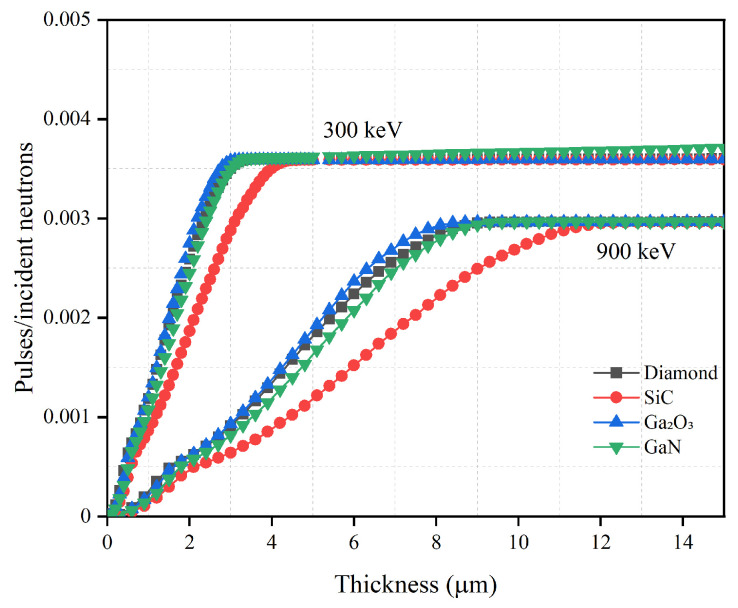
Neutron detection efficiency versus semiconductor material thickness with optimal LiF converter layer thickness for diamond, SiC, Ga${}_{2}$O${}_{3}$ and GaN semiconductor materials. The optimal converter layer thicknesses are 30.6 μm for 300 keV LLD and 27.0 μm for 900 keV LLD, as shown in Table 1.

The optimization of the semiconductor layer thickness is important for achieving the high detection efficiency while maintaining a thin semiconductor layer in order to reduce costs. Using the optimal converter layer thickness and continuously increasing the thickness of the semiconductor layer, the relationship between the thickness of different semiconductor materials and the detection efficiency is studied and is shown in Figure 3 and Figure 4. The specific optimized thickness values are shown in Table 4 and Table 5.

It can be seen from Figure 3 and Figure 4 that the detection efficiencies of different semiconductor materials first rise and then asymptote toward a constant detection efficiency. If the thickness of the converter layer is fixed, the number of charged particles created in the converter layer and absorbed by the converter layer can be determined. Therefore, the change in the detection efficiencies is mainly related to the change in the semiconductor layer thickness. When the semiconductor is thin, most charged particles lose part of their energy in this layer and then escape. As the semiconductor layer thickness increases, more energy will deposit in the semiconductor. Eventually a thickness is reached, where all charged particles will deposit all energy in the semiconductor; this thickness is considered as the optimal thickness. Increasing the semiconductor layer thickness beyond this optimal thickness does not further increase the detection efficiency.

When the converter layer material is B${}_{4}$C, the relationships between the thickness of the semiconductor layer and the detection efficiency are given in Figure 3. Of the optimized thicknesses, diamond is the thinnest at 0.7 μm for 300 keV LLD and 1.6 μm for 900 keV LLD, which shows that diamond has the strongest blocking ability to charged particles. SiC offers the second thinnest semiconductor layer, while the optimized thicknesses of SiC, GaN and Ga${}_{2}$O${}_{3}$ are very similar. Despite their elemental differences, both SiC and Ga${}_{2}$O${}_{3}$ have similar charged particle blocking capabilities because they have the same optimized thickness for 900 keV LLD with B${}_{4}$C.

When the converter layer material is LiF, the maximum intrinsic detection efficiency of four materials is significantly different to that compared with B${}_{4}$C; reduced by approximately 46% for 300 keV LLD and increased by approximately 20% for 900 keV LLD. Given this change in intrinsic detection efficiency with LiF, B${}_{4}$C is a superior converter layer when using low energy LLD for neutron detection. Due to its strong blocking ability against charged particles generated by ${}^{6}$Li(n,t)${}^{4}$He, Ga${}_{2}$O${}_{3}$ has the thinnest optimized thickness. The optimized thickness for GaN is similar to that of diamond at 3.6 μm for 300 keV LLD and 10.2 μm for 900 keV LLD. This may be related to their similar blocking ability against higher energy charged particles. The optimized thickness for SiC is the thickest of the materials simulated at 4.6 μm for 300 keV LLD and 13.2 μm for 900 keV LLD.

### 3.2. Gamma Rejection

**Figure 5:**
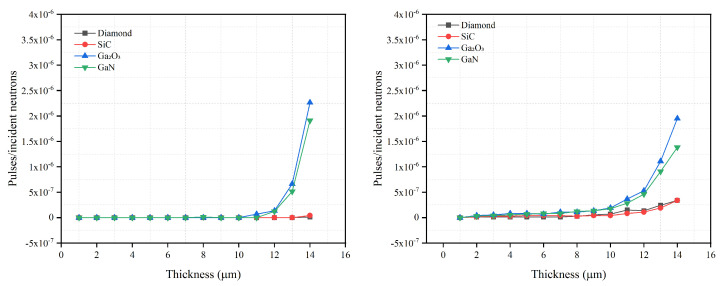
Gamma–ray detection efficiency to 511 keV photons (**left**), to 1460 keV photons (**right**) versus semiconductor thickness for 300 keV LLD with optimal B${}_{4}$C converter layer thickness for diamond, SiC, Ga${}_{2}$O${}_{3}$ and GaN semiconductor materials. The optimal B${}_{4}$C converter layer thickness is 2.6 μm for 300 keV LLD, as shown in Table 1.

**Figure 6:**
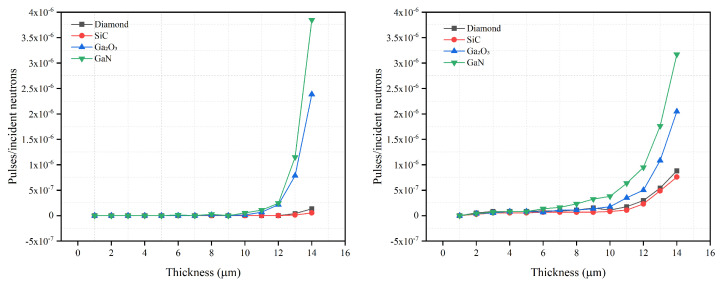
Gamma–ray detection efficiency to 511 keV photons (**left**), to 1460 keV photons (**right**) versus semiconductor thickness for 300 keV LLD with optimal LiF converter layer thickness for diamond, SiC, Ga${}_{2}$O${}_{3}$ and GaN semiconductor materials. The optimal B${}_{4}$C converter layer thickness is 30.6 μm for 300 keV LLD, as shown in Table 1.

In addition to the optimization of the semiconductor thickness, the gamma rejection capabilities of the four semiconductor materials were studied. Figure 5 and Figure 6 show the intrinsic photon detection efficiency versus the semiconductor thickness for 300 keV LLD for the two converter layers.

The intrinsic gamma detection efficiency for semiconductor materials should be less than 10${}^{-6}$ [3] in order to avoid ‘false-positive’ neutron counts. When the thicknesses of the four semiconductor materials studied are optimized, their intrinsic gamma detection efficiencies are all less than 10${}^{-6}$. Hence, all four materials meet the basic requirements for gamma rejection.

Whether SiC uses a B${}_{4}$C converter layer or a LiF converter layer, its gamma intrinsic detection efficiency is lower than that of diamond, Ga${}_{2}$O${}_{3}$ and GaN when they have the same thickness, which shows that the converter layer material will not affect the gamma rejection capability of SiC significantly. When the photon energy is 511 keV, the intrinsic gamma detection efficiency for SiC does not increase rapidly as the thickness increases. However, when the photon energy is 1460 keV, the intrinsic gamma detection efficiency for SiC increases rapidly, relative to the lower energy gamma rays, for thicknesses >11 μm. This shows that SiC is less sensitive to lower energy gamma rays.

For diamond, its gamma rejection ability is similar to that of SiC. However, the intrinsic gamma detection efficiency of the diamond detector for 1460 keV gammas increases from 0.35 × 10${}^{-6}$ with B${}_{4}$C as the converter layer to 0.75 × 10${}^{-6}$ with LiF as the converter layer, a 114% increase. This shows that the diamond neutron detector using B${}_{4}$C as the converter layer has a better gamma rejection capability than diamond using LiF. However, this observation is less noticeable for 511 keV gamma simulations. Moreover, for the different photon energies, the diamond intrinsic gamma detection efficiency is similar to that of SiC. Diamond has better gamma rejection ability under lower energy gamma rays. When the gamma ray energy is 1406 keV and the diamond thickness is >10 μm, the intrinsic gamma detection efficiency of the diamond rises rapidly, indicating a poorer gamma rejection capability.

As the thickness increases for Ga${}_{2}$O${}_{3}$, the value and growth rate of the intrinsic gamma detection efficiency does not change much for the two converter layer materials. This shows that, for the converter layer materials studied, they have little effect on the gamma rejection ability of Ga${}_{2}$O${}_{3}$. Similarly, the gamma intrinsic detection efficiency of Ga${}_{2}$O${}_{3}$ changes little under the influence of different gamma energies. It appears that gamma energy also has a limited effect on the gamma rejection ability of Ga${}_{2}$O${}_{3}$. In general, the gamma rejection capability of Ga${}_{2}$O${}_{3}$ is least affected by the converter layer material and gamma energy.

The gamma intrinsic detection efficiency of GaN is greatly affected by both the converter layer and gamma energy. When comparing the maximum intrinsic gamma detection efficiency of 14 μm thick GaN for the two different converter layers, LiF achieved a ∼3.5 × 10${}^{-6}$ average efficiency across the two energies simulated compared with ∼1.6 × 10${}^{-6}$ for B${}_{4}$C. A comparable separation in detection efficiency for GaN with LiF versus B${}_{4}$C was observed for GaN layer thicknesses >10 μm. When the converter layer material is B${}_{4}$C, the intrinsic gamma detection efficiency of 14 μm GaN under 511 keV and 1460 keV gamma irradiation is 1.91 × 10${}^{-6}$ and 1.38 × 10${}^{-6}$, respectively. When the converter layer material is changed to LiF, the intrinsic gamma detection efficiency of 14 μm GaN is 3.85 × 10${}^{-6}$ and 3.17 × 10${}^{-6}$ (2 d.p.) for 511 keV and 1460 keV energies, respectively. The difference in the intrinsic gamma detection efficiency of 14 μm GaN with different converter layers is approximately 45% and 56%. On the other hand, gamma rays of different energies will affect the growth rate of the intrinsic gamma detection efficiency of GaN as the thickness increases. Nevertheless, it is worth noting that higher energy gamma rays will increase the intrinsic gamma detection efficiency of GaN at lower thicknesses (<10 μm). However, when GaN exceeds 10 μm, its intrinsic detection efficiency for lower energy gamma rays is greater than that for higher energy gamma rays. The reason is that, when the thickness of GaN is low, the gamma rays of 511 keV easily escape the GaN. On the other hand, in addition to Compton scattering, due to the higher energy of the 1460 keV gamma rays, the number of electron pair effects increases, improving gamma detection efficiency. When the thickness of the GaN is increased to a certain thickness, the gamma rays of 511 keV can easily deposit more energy in the GaN, such that the intrinsic detection efficiency of the GaN to the 511 keV gamma rays increases. In summary, compared to the LiF converter layer, the GaN neutron detector with the B${}_{4}$C converter layer has a better gamma rejection capability. Moreover, because the optimized thickness of the GaN semiconductor layer is less than 3 μm with the B${}_{4}$C converter layer, it has a stronger rejection capability for lower energy gamma rays.

### 3.3. Radiation Hardness

**Figure 7:**
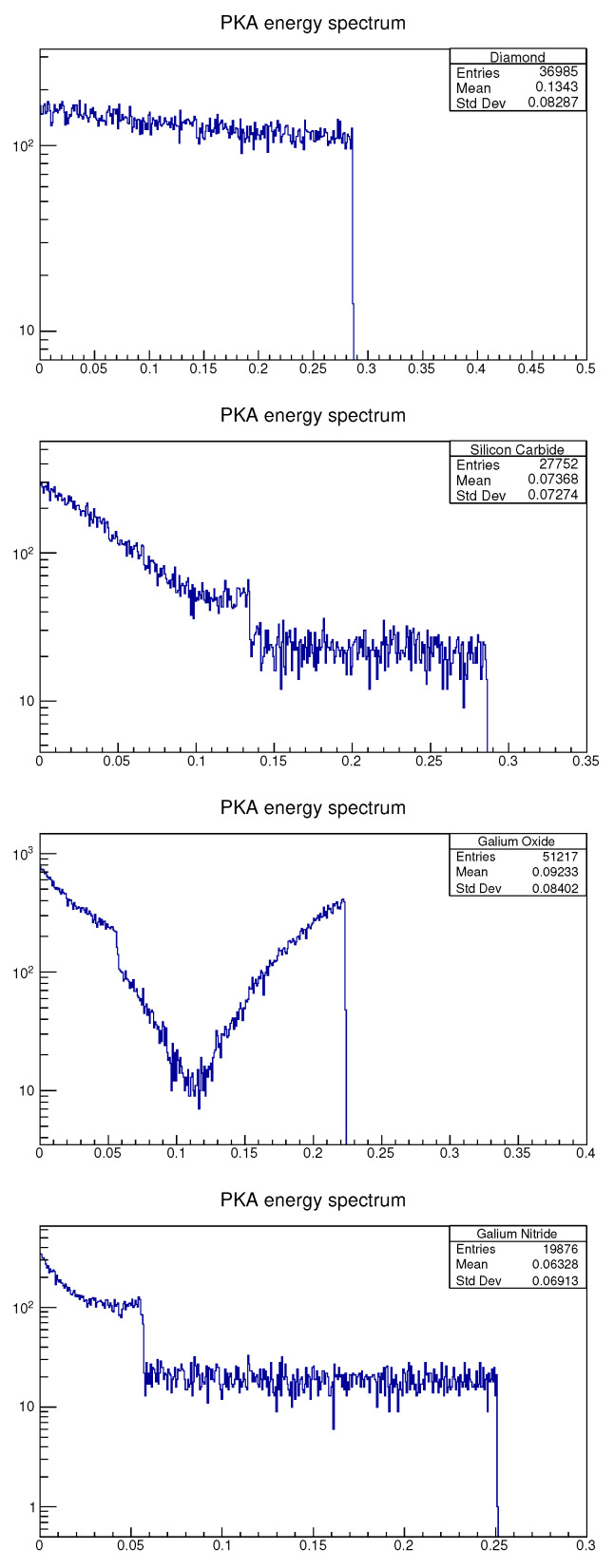
Primary knock-on atoms (PKA) energy spectra for diamond, SiC, Ga${}_{2}$O${}_{3}$ and GaN. These energy spectra were created by ROOT. The X-axis shows the energy of PKA in MeV. The Y-axis shows the count number of PKA with a certain energy. The figure keys show: Entries, the total number of PKA; Mean, the average PKA energy in MeV; and Std Dev, the standard deviation of PKA energy (MeV).

One of the reactions between neutrons and a nucleus is elastic scattering. There is a chance that the nucleus will be knocked out of the lattice by an incident neutron that has an energy of up to approximately 1 MeV. The simulated PKA energy spectrum for the four materials modelled are shown in Figure 7. The atomic displacements are then calculated using (3). The DPA is then calculated by dividing the atomic displacements by the total number of atoms in the material. The smaller the DPA, the less likely the atoms are to leave the lattice position. This can be used as a measure of the radiation hardness of the material. In reality, there is a possibility that an atom may return to the lattice position after being knocked out by a neutron. However, DPA still has significance for the qualitative analysis, performed in this study, of the radiation hardness of materials. The unit of the DPA value is DPA × ${\mathrm{cm}}^{2}$/incident particles [38].

Table 6 shows the calculated DPA and average PKA energy (keV).

### 3.4. Electron Collection Efficiency

In this section, the electron collection efficiency of four material with optimal thickness are studied.

**Figure 8:**
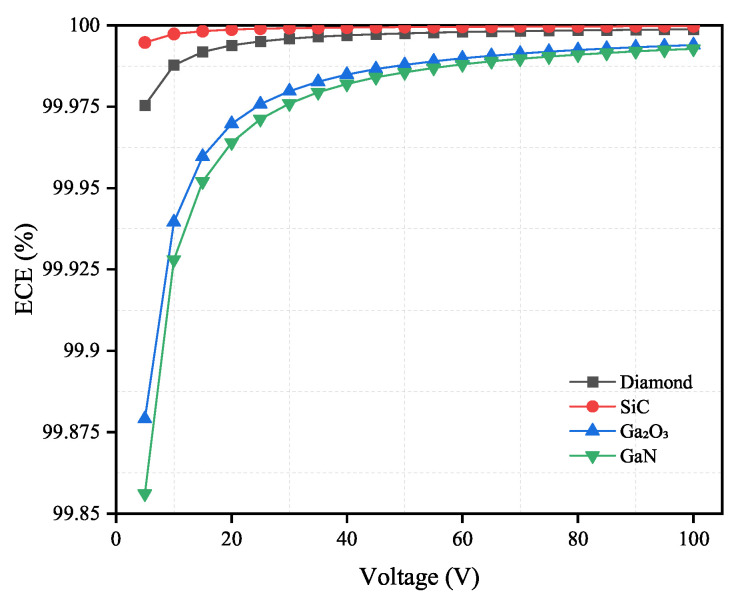
Electron collection efficiency (%) versus applied voltage for diamond, SiC, Ga${}_{2}$O${}_{3}$ and GaN semiconductor materials. The thickness for four materials are shown in Table 4.

**Figure 9:**
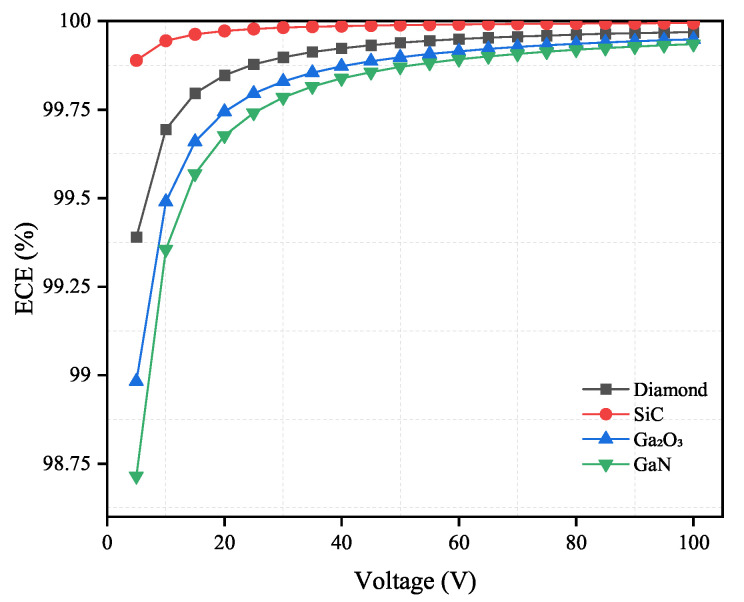
Electron collection efficiency (%) versus applied voltage for diamond, SiC, Ga${}_{2}$O${}_{3}$ and GaN semiconductor materials. The thickness for four materials are shown in Table 5.

As shown in Figure 8 and Figure 9, the ECE will increase as the applied voltage increases. When the electron mobility and carrier lifetime of materials are fixed, the ECE will decrease with a thicker material.

## 4. Discussion

For thickness optimisation, after comparison, diamond is found to perform very well for the two converter layer materials, and the optimized thickness is small. When GaN is combined with a B${}_{4}$C converter layer, the optimized thickness is greater than that of diamond (1.2 μm and 2.4 μm for GaN compared with 0.7 μm and 1.6 μm for diamond). However, when combined with LiF, the optimized thickness and intrinsic detection efficiency for GaN and diamond are similar: 3.6 μm for 300 keV LLD and 10.2 μm for 900 keV LLD. However, for GaN with a LiF converter layer, the detection efficiency compared to that of GaN with a B${}_{4}$C converter layer is nearly 50% lower. Therefore, for GaN detectors, B${}_{4}$C is more suitable as a converter layer than LiF. When the converter layer is LiF, the optimized thickness of SiC is much greater than that of the other three materials. Therefore, B${}_{4}$C is the superior choice for SiC neutron detectors. Furthermore, considering both the growth of SiC semiconductor devices and the fact that the manufacturing processes are more mature, the suitability and performance of SiC will be better accessed with a study that accounts for an external electric field. Ga${}_{2}$O${}_{3}$ has a surprising performance when combined with LiF: the optimized thickness is the lowest of four materials. Due to the fact that it is easier to obtain LiF than B${}_{4}$C, especially for B${}_{4}$C with a high boron purity, Ga${}_{2}$O${}_{3}$ is the best choice of these four materials with a LiF converter layer. However, considering detection efficiency, a B${}_{4}$C converter layer is the better choice for Ga${}_{2}$O${}_{3}$, as it will have a lower semiconductor thickness and higher detection efficiency than with LiF as the converter layer.

Diamond and SiC have better gamma rejection capabilities than Ga${}_{2}$O${}_{3}$ and GaN. In this study, when facing different converter layer materials and gamma ray energies, the intrinsic gamma detection efficiency of diamond and SiC thinner than 14 μm are both less than 10${}^{-6}$. Specifically, diamond and SiC with a B${}_{4}$C converter layer have better gamma rejection capabilities than with a LiF converter layer. This phenomenon is more apparent when the thickness of the diamond or SiC is greater than 10 μm. The intrinsic gamma detection efficiency of Ga${}_{2}$O${}_{3}$ is also less affected by the converter layer material and gamma energy. However, compared with SiC and diamond, when the thickness of Ga${}_{2}$O${}_{3}$ exceeds 10 μm, its intrinsic gamma detection efficiency will quickly exceed 10${}^{-6}$. For GaN, a detector using B${}_{4}$C as the converter layer has a better gamma rejection capability than that using LiF as the converter layer.

The materials in ascending DPA order are GaN, diamond, SiC and Ga${}_{2}$O${}_{3}$. This indicates that GaN has the best radiation hardness when subjected to a 1 MeV neutron flux. Furthermore, GaN has the smallest average PKA energy, which means that GaN will have less SKA that cause displacements. In this research, only the PKA energy spectrum is simulated, and the DPA is calculated using the PKA. However, PKA will sometimes create SKA that, in turn, can cause atomic displacements too. The material with the lowest average PKA energy, GaN, would suggest that it has the lowest SKA. However, follow-up molecular dynamics research would be required to scrutinize this hypothesis. The DPA of the diamond is lower than that of SiC and Ga${}_{2}$O${}_{3}$, despite the average PKA energy of diamond being higher than that of SiC and Ga${}_{2}$O${}_{3}$. This demonstrates that PKA in diamond will create fewer atomic displacements than SiC and Ga${}_{2}$O${}_{3}$, but diamond may create more SKA than SiC and Ga${}_{2}$O${}_{3}$.

According to the results as shown in Figure 8 and Figure 9, both diamond and SiC have high ECE under the applied voltage from 5 V to 100 V. This is because diamond has the fastest electron mobility and SiC has the longest electron lifetime among the four materials. This demonstrates that the change in applied voltage has limited influence on these two materials. Diamond and SiC can theoretically be used to fabricate low-bias or even zero-bias voltage neutron detectors. For Ga${}_{2}$O${}_{3}$ and GaN, the change in the applied voltage affects their ECE significantly. However, when the applied voltage reaches 45 V or higher, the influence of the applied voltage becomes relatively small. Therefore, in practical applications, 45 V can be used as the operating voltage of the detector.

## 5. Conclusions

This research investigated the optimal thicknesses, the gamma rejection performance, the PKA, DPA and the ECE of four semiconductor materials using two different converter layers.

The materials in ascending optimal thickness order are diamond, SiC, Ga${}_{2}$O${}_{3}$ and GaN with a B${}_{4}$C converter layer and Ga${}_{2}$O${}_{3}$, diamond, GaN and SiC with a LiF converter layer. Overall, the diamond with the B${}_{4}$C converter layer has the best performance in thickness optimization, gamma rejection capability, radiation hardness and charge collection. Despite methods such as metal–organic chemical vapor deposition (MOCVD) and molecular beam epitaxy (MBE) for growing diamond crystals, the main limitation of diamond is still the cost. If the cost of diamond can be significantly reduced, diamond neutron detectors will be a good candidate for commercial applications. Considering the cost, SiC with B${}_{4}$C is the best choice for commercial applications due to its thin optimal thickness, good gamma rejection capability, charge collection and relative mature manufacturing technology compared with the other three materials. GaN has the best performance in the radiation hardness of these four materials. Compared with LiF, B${}_{4}$C is the better choice for GaN because it has a thinner optimized thickness, better gamma rejection capability and high ECE. Besides, other limitations of GaN are not limited to the crystal quality, especially for the demand of a high-quality p-type GaN, high charger doping concentration and longer carrier lifetime, which will reduce the GaN radiation hardness, neutron detection efficiency and CCE. On the other hand, with more research on improving the quality of GaN [27], it is a semiconductor material with great potential for neutron detection under extreme environments. Ga${}_{2}$O${}_{3}$ has the widest band gap (4.9 eV) of these four materials. Moreover, Ga${}_{2}$O${}_{3}$ has the lowest optimized thickness of the four materials when the converter layer is LiF. Nevertheless, Ga${}_{2}$O${}_{3}$ performed the second worst in terms of its gamma rejection capability with LiF as the converter layer.

In summary, this study optimized four thin-film semiconductor neutron detectors and demonstrated their intrinsic neutron detection efficiency, gamma rejection capability, neutron irradiation response and electron charge collection in an effort to advance research on novel neutron detectors for use in harsh environments. Future studies will focus on further radiation damage simulation and its effects on the CCE of these materials.

## Figures and Tables

**Table 1:** Optimal converter layer thicknesses for B${}_{4}$C and LiF [30].

LLD	B${}_{4}$C	LiF
300 keV	2.6 μm	30.6 μm
900 keV	1.7 μm	27.0 μm

**Table 2:** Threshold displacement energy for diamond, 4H-SiC, Ga${}_{2}$O${}_{3}$ and GaN. Reference to source is shown in square parentheses.

	Diamond	4H-SiC	Ga${}_{2}$O${}_{3}$	GaN
Threshold displacement energy (eV)	35 [4]	Si:35 C:20 [4]	25 [32]	Ga:18 N:22 [4]

**Table 3:** Electron mobility and electron lifetime for diamond, 4H-SiC, Ga${}_{2}$O${}_{3}$ and GaN. Reference to source is shown in square parentheses.

	Diamond	4H-SiC	Ga${}_{2}$O${}_{3}$	GaN
Electron mobility μ (${\mathrm{cm}}^{2}$/V·s) [33]	≈2000	≈1000	≈300	≈1200
Electron lifetime τ (μs)	≈2 [34]	≈19 [35]	≈1 [36]	≈1 [37]

**Table 4:** Optimal semiconductor thickness with optimal B${}_{4}$C converter layer thickness for diamond, SiC, Ga${}_{2}$O${}_{3}$ and GaN semiconductor materials. The optimal converter layer thicknesses are 2.6 μm for 300 keV LLD and 1.7 μm for 900 keV LLD, as shown in Table 1.

LLD	B${}_{4}$C	Diamond	SiC	Ga${}_{2}$O${}_{3}$	GaN
300 keV	2.6 μm	0.7 μm	1.0 μm	1.1 μm	1.2 μm
900 keV	1.7 μm	1.6 μm	2.2 μm	2.2 μm	2.4 μm

**Table 5:** Optimal semiconductor thickness with optimal LiF converter layer thickness for diamond, SiC, Ga${}_{2}$O${}_{3}$ and GaN semiconductor materials. The optimal converter layer thicknesses are 30.6 μm for 300 keV LLD and 27.0 μm for 900 keV LLD, as shown in Table 1.

LLD	LiF	Diamond	SiC	Ga${}_{2}$O${}_{3}$	GaN
300 keV	30.6 μm	3.5 μm	4.6 μm	3.2 μm	3.6 μm
900 keV	27.0 μm	9.9 μm	13.2 μm	9.3 μm	10.2 μm

**Table 6:** Displacements per atom and average primary knock-on atoms energy of four materials under a 1 MeV neutron flux.

	Diamond	SiC	Ga${}_{2}$O${}_{3}$	GaN
DPA (×10${}^{-20}$)	1.30	4.12	6.83	1.01
Average PKA energy (keV)	134.30	73.68	92.33	63.28

## Abbreviations

The following abbreviations are used in this manuscript:

PKA	Primary knock-on atom
SKA	Secondary knock-on atom
DPA	Displacement per atom
CCE	Charge collection efficiency
ECE	Electron collection efficiency
